# *Erwinia amylovora* CRISPR Elements Provide New Tools for Evaluating Strain Diversity and for Microbial Source Tracking

**DOI:** 10.1371/journal.pone.0041706

**Published:** 2012-07-31

**Authors:** Gayle C. McGhee, George W. Sundin

**Affiliations:** Department of Plant Pathology and Centers for Microbial Ecology and Pathogenesis, Michigan State University, East Lansing, Michigan, United States of America; University of Wisconsin-Milwaukee, United States of America

## Abstract

Clustered regularly interspaced short palindromic repeats (CRISPRs) comprise a family of short DNA repeat sequences that are separated by non repetitive spacer sequences and, in combination with a suite of Cas proteins, are thought to function as an adaptive immune system against invading DNA. The number of CRISPR arrays in a bacterial chromosome is variable, and the content of each array can differ in both repeat number and in the presence or absence of specific spacers. We utilized a comparative sequence analysis of CRISPR arrays of the plant pathogen *Erwinia amylovora* to uncover previously unknown genetic diversity in this species. A total of 85 *E. amylovora* strains varying in geographic isolation (North America, Europe, New Zealand, and the Middle East), host range, plasmid content, and streptomycin sensitivity/resistance were evaluated for CRISPR array number and spacer variability. From these strains, 588 unique spacers were identified in the three CRISPR arrays present in *E. amylovora*, and these arrays could be categorized into 20, 17, and 2 patterns types, respectively. Analysis of the relatedness of spacer content differentiated most apple and pear strains isolated in the eastern U.S. from western U.S. strains. In addition, we identified North American strains that shared CRISPR genotypes with strains isolated on other continents. *E. amylovora* strains from *Rubus* and Indian hawthorn contained mostly unique spacers compared to apple and pear strains, while strains from loquat shared 79% of spacers with apple and pear strains. Approximately 23% of the spacers matched known sequences, with 16% targeting plasmids and 5% targeting bacteriophage. The plasmid pEU30, isolated in *E. amylovora* strains from the western U.S., was targeted by 55 spacers. Lastly, we used spacer patterns and content to determine that streptomycin-resistant strains of *E. amylovora* from Michigan were low in diversity and matched corresponding streptomycin-sensitive strains from the background population.

## Introduction

The enterobacterial pathogen *Erwinia amylovora* is the causal agent of fire blight, a devastating disease of apple, pear, and other plants in the Rosaceae family. The bacterium initiates infection of flowers following colonization of intercellular spaces on stigmas and growth to relatively large population sizes (ca. 10^5^ to 10^6^ cfu/flower). Following flower infection, *E. amylovora* invades the plant and migrates internally producing biofilms within the plant vascular system, and can also emerge as bacterial ooze (cells embedded in exopolysaccharide) that can be transmitted to and infect other nearby plants [1], [2]. Management of fire blight is difficult, and is exacerbated by the development of streptomycin resistance in *E. amylovora* populations [3].

The *E. amylovora* species exhibits very low levels of genetic diversity. For example, comparison of the complete genome sequences of two *E. amylovora* strains isolated from apple and pear on different continents revealed 99.99% sequence identity [4], [5]. In addition, other typing methods, based on pulsed field gel electrophoresis, REP-PCR fingerprinting, ribotyping, and variable number of tandem repeat analyses revealed the difficulties in accurately and effectively differentiating strains [6], [7], . Only *E. amylovora* strains isolated from *Rubus* spp. are sufficiently diverged from the apple and pear genotypes to enable easy molecular differentiation [12], [13]. *E. amylovora* is also closely related to other tree fruit pathogens *E. pyrifoliae* and *Erwinia* sp. isolated from pear in Asia and to the nonpathogenic species *E. billingiae* and *E. tasmaniensis* [14], [15], [16].

**Table 1:** *Erwinia amylovora, E. pyrifoliae*, and *Erwinia* sp. strains used in this study and their relevant characteristics, including sensitivity/resistance to streptomycin and streptomycin resistance genotype.

Strain	Geographic location	Host	Year	Known plasmids	Streptomycin Sensitivity/location of *strAB* genes	Ribo-type^a^	groEL seq type^b^
**Midwest, Eastern US and Canada**							
BH	Hart, MI	Apple	2008	pEA29	Sensitive		1
DP11	Michigan	Pear	1993	pEA29	Sensitive	1	1
DR5	*Michigan*	Apple	1993	pEA29	Sensitive	1	1
Ea(T1)2	Michigan	Apple	1997	pEA29	Sensitive		1
Ea(T3)2	Michigan	Apple	1997	pEA29	Sensitive		1
Ea110	Ingham County, MI	Apple	1975	pEA29	Sensitive		1
EL01	East Lansing, MI	Apple	1993	pEA29	Sensitive	1	1
GH9	Michigan	Apple	1993	pEA29	Sensitive	1	1
K2	Michigan	Apple	1993	pEA29	Sensitive	1	1
L14	Michigan	Apple	1993	pEA29	Sensitive	1	1
MK1	Michigan	Apple	1993	pEA29	Sensitive	1	1
NW17-4	Northwest MI	Apple	2011	pEA29	Sensitive		1
Pn	Hart, MI	Apple	2008	pEA29	Sensitive		1
RB02	Michigan	Apple	1993	pEA29	Sensitive	3	1
RB07	Michigan	Apple	1993	pEA29	Sensitive	1	1
RL3	Michigan	Apple	1993	pEA29	Sensitive	1	1
RRP12	Michigan	Apple	1993	pEA29	Sensitive	1	1
BBA-8	Southwest MI	Apple	2007	pEA29	MR, pEA29 bp 17527		1
BCN20	Southwest MI	Apple	1995	pEA29	MR, pEA29 bp 12360	1	1
CA11	Southwest MI	Crab apple	1993	pEA29, pEA34	MR, pEA34	1	1
DM1	Southwest MI	Apple	1994	pEA29	MR, pEA29 bp 17527	1	1
EaRoo29	Southwest MI	Apple	1997	pEA29	MR, pEA29 bp 17527 and pEA34		1
GR5B1	Grand Rapids, MI	Apple	2004	pEA29, pEA34	MR, pEA29 bp 1515 and pEA34		1
HS10	Southwest MI	Apple	1993	pEA29, pEA34	MR, pEA34	1	1
KL	Ionia County, MI	Apple	2008	pEA29	MR, pEA29 bp 1515		1
KR	Ionia County, MI	Apple	2007	pEA29	MR, pEA29 bp 1515		1
MA-1	Southwest MI	Apple	2007	pEA29	MR, pEA29 bp 1515		1
MC-5	Southwest MI	Apple	2007	pEA29	MR, pEA29 bp 1515		1
MI5-1	Southwest MI	Apple	2002	pEA29	MR, pEA29 bp 1515		1
NW H26	Northwest MI	Apple	2011	pEA29	MR, pEA29 bp 1515		1
NW1-1	Northwest MI	Apple	2011	pEA29	MR, pEA29 bp 17527		1
NW18-6	Northwest MI	Apple	2011	pEA29	MR, pEA29 bp 1515		1
NW2-11	Northwest MI	Pear	2011	pEA29	MR, pEA29 bp 1515		1
NW21-4	Northwest MI	Pear	2011	pEA29	MR, pEA29 bp 1515		1
NW2A	Northwest MI	Pear	2011	pEA29	MR, pEA29 bp 1515		1
NW3-1	Northwest MI	Apple	2011	pEA29	MR, pEA29 bp 17527		1
RA	Grand Rapids, MI	Apple	2005	pEA29	MR, pEA29 bp 1515		1
RM5	Southwest MI	Apple	2007	pEA29	MR, pEA29 bp 17527		1
S5	Southwest MI	Apple	1994	pEA29	HR, mutation *rpsL*		1
SB1-9	Southwest MI	Apple	2003	pEA29	MR, pEA29 bp 17527		1
W4	Southwest MI	Apple	2007	pEA29	MR, pEA29 bp 17527		1
Ea273	New York	Apple	1971	pEA29	Sensitive		1
6–97	Canada	Apple	2000	pEA29	Sensitive		1
**Western USA**							
87–70	Washington State	Apple		pEA29	Sensitive	1	1
87–73	Washington State	Apple		pEA29	Sensitive	1	1
Ca1R	California	Apple	1995	pEA29	Sensitive	3	2
Ca3R	California	Apple	1995	pEA29	MR	1	1
Ea88	Washington State	Pear	1995	pEA29	HR, mutation *rpsL*	3	2
FB93-9	Idaho	Apple		pEA29	Sensitive	1	1
JL1189	Washington	Pear	1988	pEA29, pEU30	HR, mutation in *rpsL*	3	2
LA004	Washington	Pear		pEA29, pEU30	HR, mutation in *rpsL*	3	2
La092	Washington	Pear	1988	pEA29, pEU30	HR, mutation in *rpsL*	3	2
LP101	Washington	Apple		pEA29	Sensitive	3	2
OR1	Oregon	Pear		pEA29	HR, mutation in *rpsL*	3	2
OR6	Oregon	Pear		pEA29	HR, mutation in *rpsL*	3	2
UT5P4	Utah	Apple	2000	pEA29, pEU30	Sensitive		1
UTFer3	Utah	Pear	2000	pEA29	HR, mutation in rpsL		1
UTRJ2	Utah	Pear	2000	pEA29, pEU30	HR, mutation in *rpsL*		1
WSDA 16	Washington	Apple		pEA29	Sensitive		1
**Europe, Middle East and New Zealand**							
1596	Spain	unknown		pEA70 only	Sensitive		1
B3	Serb/Montenegro	unknown		No plasmids	Sensitive		1
CFBP1430	France	Hawthorne	1972	pEA29	Sensitive		1
Ea1189	Germany	unknown		pEA29	Sensitive		1
Ea322	France	Pear		pEA29, pCPP60	Sensitive		1
Leb A-1	Lebanon	Pear	1998	pEA29, pEL60	HR, mutation *rpsL*		1
Leb A-16	Lebanon	Pear	1998	pEA29, pEL60	HR, mutation *rpsL*		1
Leb A-19	Lebanon	Pear	1998	pEA29	Sensitive		1
Leb A-3	Lebanon	Quince	1998	pEA29, pEL60	Sensitive		1
Leb B-66	Lebanon	Apple	1998	pEA29, pEL60	HR, mutation *rpsL*		1
NZR3	New Zealand	unknown	1992	pEA29	HR, mutation in *rpsL*	1	1
NZR5	New Zealand	unknown	1992	pEA29	HR, mutation in *rpsL*	1	1
NZS24	New Zealand	unknown	1992	pEA29	Sensitive	1	1
OT-1	England	unknown		pEA29	Sensitive		1
***E. amylovora*** ** from ** ***Rubus*** ** USA**							
IL5	Illinois	Raspberry	1977	pEA29, small plamids	Sensitive	4	1
IL6	Illinois	Raspberry	1977	pEA29, small plasmids	Sensitive	4	1
MR1	Alcona Co., MI	Raspberry	1995	pEA29	Sensitive	4	3
OKR1	Oklahoma	Raspberry	2002	pEA29	Sensitive		1
RBA4	Alpena Co., MI	Raspberry		pEA29	Sensitive	4	3
Rkk3	Michigan	Raspberry		pEA29	Sensitive	4	3
***E. amylovora*** ** from Indian Hawthorn**							
IH2-3	South Carolina	Indian Hawthorn	1998	pEA29, small plasmids	Sensitive		1
IH3-1	South Carolina	Indian Hawthorn	1998	pEA29, small plasmids	Sensitive		
***E. amylovora*** ** from Loquat**							
TxLo3	Texas	Loquat	2011	pEA29	Sensitive		2
TxLo4	Texas	Loquat	2011	pEA29	Sensitive		2
TxLo6	Texas	Loquat	2011	pEA29	Sensitive		2
TxLo7	Texas	Loquat	2011	pEA29	Sensitive		2
**Other Erwinias**							
*E. pyrifoliae* Ep1/96	Korea	*Pyrus pyrifolia*	1996	pEP36, small plasmids			5
*E. pyrifoliae* Ep4/97	Korea	*Pyrus pyrifolia*	1997	pEP36, small plasmids			5
*E. pyrifoliae* Ep16/96	Korea	*Pyrus pyrifolia*	1996	pEP36, small plasmids			5
Erwinia spp. Ejp617	Japan	Nashi pear,	1996	pEJ30, small plamids			4
Erwinia spp. Ejp556	Japan	Nashi pear,	1994	pEJ30, small plamids			
Erwinia spp. Ejp557	Japan	Nashi pear,	1994	pEJ30, small plamids			

a S  =  sensitive, minimum inhibitory concentration (MIC) <100 µg ml^−1^, MR  =  medium resistance MIC between 100 µg ml^−1^and 1000 µg ml^−1^, and HR =  MIC >2000 µg ml^−1^. Streptomycin phenotypes and MICs for some strains have been reported previously.

b Transposon Tn*5393* harboring *strA* and *strB* resistance genes present on plasmid pEA29 at bp 1515 (29–1), bp 17527 (29–2) or bp 12360 (29–3), or on conjugative plasmid pEA34. *rpsL*  =  high level of resistance caused by a mutation in *rpsL* gene [56].

c Ribotyping previously reported in [8].

d Four hundred and nine bp of the *groEL* gene was amplified and sequenced using primers groEL-A and groEL-B. Pattern 1 is the predominant pattern and is found in sequenced strains ATCC 49942 and CFBP1430. Pattern 2 has 2 nucleotide changes from a C to a T at bp 329 and 335; Pattern 3 has a single bp change of T to C at bp 299. Pattern 4 has 18 bp changes at positions 50, 53, 134, 140, 146, 152, 155, 215, 228, 281, 299, 302, 314, 341, 374, 413, 422, and 437. Pattern 5 is similar to pattern 4 except that there is an additional bp change at position 203.

e TS  =  This study.

Information on the presence and characterization of laterally-acquired DNA sequences and indigenous plasmids in *E. amylovora* has been reported in a few studies. Almost all *E. amylovora* strains contain a plasmid of approximately 29 kb termed the ubiquitous plasmid pEA29 [17], [18]. This nonconjugative plasmid plays a role in virulence linked to the carriage of thiamin-biosynthetic genes [19]. In addition to pEA29, variation in plasmid profiles or lack of pEA29 has been used to differentiate strains, although the contribution of other plasmids detected in *E. amylovora* to virulence has not been demonstrated [20], [21], [22], [23], except for the recently described pEI70 which affects aggressiveness in an immature pear infection model [24]. However, the presence of different plasmid patterns in various strains indicates that the *E. amylovora* species has been subject to plasmid invasion and colonization during its life history. Streptomycin-resistant (Sm^R^) strains of *E. amylovora* isolated in Michigan harbor the transposon Tn*5393* that encodes the streptomycin-resistance genes *strA-strB* [25], [26]. This transposon was thought to be obtained by *E. amylovora* from the orchard epiphyte *Pantoea agglomerans* on the plasmid pEa34 [25]. A subsequent genetic analysis recently demonstrated that only very few strain types are responsible for the dissemination of streptomycin resistance in Michigan, and that Tn*5393* had moved to pEA29 in these strains [27]. This observation suggests that gene transfer events resulting in the acquisition of resistance genes may be relatively rare in the *E. amylovora* population. Finally, there have been a few reports including some sequence availability of bacteriophage that can infect *E. amylovora* [28], [29], and experiments by Schnabel and Jones [30] established differential sensitivity of *E. amylovora* isolates from Michigan to a panel of five bacteriophage.

*E. amylovora* is thought to have originated in North America, and was first observed on apple in New York in the 1700s following the introduction of apple to the continent by European settlers [31]. Fire blight has since spread to apple and pear in over 46 countries. Epidemiology and strain tracking provides knowledge that is critical to topics such as determining outbreak centers for disease epidemics and determining origins of strains detected in countries in which *E. amylovora* is a quarantine organism. In our own studies tracking the occurrence of Sm^R^ strains of *E. amylovora* in Michigan, we were interested in determining if these strains were introduced to Michigan or originated from local strains that had acquired the Sm^R^ transposon Tn*5393*. However, successful strain tracking requires an ability to efficiently distinguish individuals, and our recent comparative sequence analysis of housekeeping genes such as *groEL* indicated an extremely low level of genetic diversity within a collection of 34 streptomycin-sensitive (Sm^S^) and Sm^R^ strains [27].

Clustered regularly interspaced short palindromic repeats (CRISPRs) comprise a family of short DNA repeat sequences found in approximately half of all bacterial and archeal genomes. These repeats range in size from 21 to 47 bp and are separated by regularly-sized nonrepetitive spacer sequences. Many of the spacer sequences associated with CRISPRs share sequence identity with bacteriophage, plasmid, and other laterally-transferred DNA sequences [32], [33]. Together, these sequences, along with a suite of Cas (CRISPR-associated) and Cse (CRISPR Cascade complex) proteins, are thought to function as an adaptive immune system that targets invading foreign DNA in a sequence-specific manner [34], [35]. Because the spacer sequences are added in a specified location at the CRISPR locus, spacer sequence arrays are temporally ordered with the oldest spacers located at the 3′ end of each array and the more recent spacer additions at the 5′ end. Thus, it has been postulated that the spacers represent a record of past encounters of the organism under study with potential invading sequences [36]. The presence of particular spacer sequences within a CRISPR locus is also evidence of the lack of success of the DNA element harboring that sequence in colonizing the organism [34]. Finally, this record of past encounters is historical, i.e. capable of changing over time, and also may include a geographic component, if isolates of the same species dwelling in distinct geographic habitats are exposed to different backgrounds of mobile DNA sequences and there is little isolate mixing between habitats. It should be noted that the geographic inference also assumes that the individual CRISPR loci under study are functional in the organism they are characterized from.

We hypothesized that *E. amylovora* strains would harbor CRISPR repeat sequences that would differ either in spacer identity and/or spacer array patterns to enable the use of this locus for strain tracking. In this study, we sequenced and analyzed CRISPR loci in a diverse collection of *E. amylovora* and related species. Our results indicate a wide diversity of spacer sequences based on geography and host of isolation and suggest that CRISPRs can be effectively utilized in population and epidemiological analyses of *E. amylovora*.

## Materials and Methods

### Bacterial Strains

The bacterial strains characterized for CRISPR array number and content for this study are listed in Table 1. Many of these strains had been characterized previously by ribotyping [8], and the plasmid content of each strain is known. In addition, the Sm^R^ phenotype, genetic mechanism of streptomycin resistance, and location of Tn*5393*, if present, is summarized in Table 1. All strains were stored in 15% glycerol at −80°C prior to use. *Erwinia amylovora* and other *Erwinia* spp. strains were maintained on LB agar and cultured at 28°C. Where necessary, streptomycin (100 or 2,000 µg ml^-1^) was added to LB for resistance screening.

**Figure 1:**
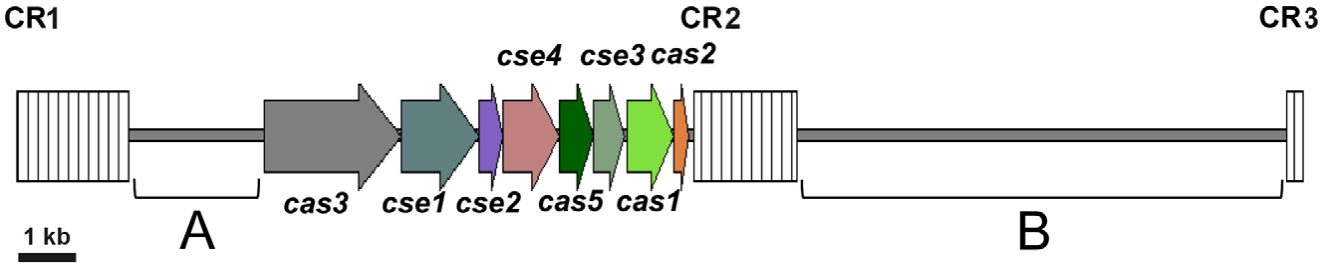
Genetic map of the CRISPR locus of *E. amylovora* ATCC 49946 showing the location of *cas* and *cse* genes and the spacer arrays CR1, CR2, and CR3. Sequences denoted by brackets and designated “A” and “B” contain housekeeping genes apparently unrelated to CRISPR function.

### DNA Extraction, Amplification, and Sequencing

Genomic DNA preps were prepared for each strain by suspending single colonies in 100 µl of a lysis buffer (0.5 M KCL, 0.01 M Tris-HCL [pH 8.5], 1% Tween 20) and boiling for 10 min. The lysate was used as a template in PCR reactions. All PCR reactions (50 µl) contained 1× PCR buffer (10 mM Tris-HCl at pH 8.3), 1.5 mM MgCl_2_, 0.2 mM of each deoxynucleotide triphosphate, 20 pM of each primer, 2.5 U of *Taq* DNA polymerase (Invitrogen; Carlsbad, CA), and 3 µl of bacterial lysate.

Oligonucleotide primers used to amplify the CRISPR 1, 2, and 3 array sequences were designed using the *E. amylovora* Ea273 (ATCC 49946) genome sequence (GenBank accession number NC_013971). Primer sequences are listed in Table S1. The cycling parameters were 94°C for 5 min followed by 40 cycles of 94°C for 30 seconds, annealing temperatures of 58°C (CRISPR 1 and 2) or 55°C (CRISPR 3) for 30 seconds, followed by 72°C for 4 min (CRISPR 1 and 2) or 45 seconds (CRISPR 3) with a final extension time for 7 min at 72°C. Amplification parameters for *E. pyrifoliae* CRISPRs 1–4 were identical to those listed above for CRISPRs 1 and 2 from isolates of *E. amylovora*.

Amplification and sequencing of a partial region of *groEL*, a housekeeping gene coding for Hsp60 commonly used to compare bacterial strains, was also reported in this study. The *groEL* gene has been previously used to compare *E. amylovora* both intra-species and inter-species to closely related *Erwinia* species. Amplification of the partial *groEL* gene from the *E. amylovora* chromosome was performed as previously described using primers groEL-A and groEL-B [12], [22], [27].

Amplified PCR products were purified using the QIAquick PCR Purification Kit (Qiagen, Valencia, CA) and sequenced. All sequencing was performed at the Michigan State University Research Technology Support Facility using ABI dye-terminator chemistry and ABI 3730 genetic analyzer (Applied Biosystems, Foster City, CA). Where necessary, primer walking was used to complete sequencing.

### CRISPR Array Analysis and Alignment

Sequences were assembled using the DNASTAR Lasergene Software Suite for Sequence Analysis Version 7.2.0 (DNASTAR, Inc.; Madison, WI). CRISPR array spacer and repeat patterns were generated using the CRISPR recognition tool (CRT) Version 1.0 (http://www.room220.com/crt) [37]. The repeat sequences within the CRISPRs 1, 2, and 3 were assessed for existing homology to known sequences using BLAST searches of the GenBank database (http://blast.ncbi.nlm.nih.gov/Blast.cgi). Alignments of repeat sequences to those present in other species were done using WebLogo (http://weblogo.berkeley.edu/logo.cgi). Each spacer in the array was also assessed for homology to known sequences using BLASTn. All spacers were compared among strains to identify unique spacers and to identify conserved patterns of spacers among strains.

**Figure 2:**
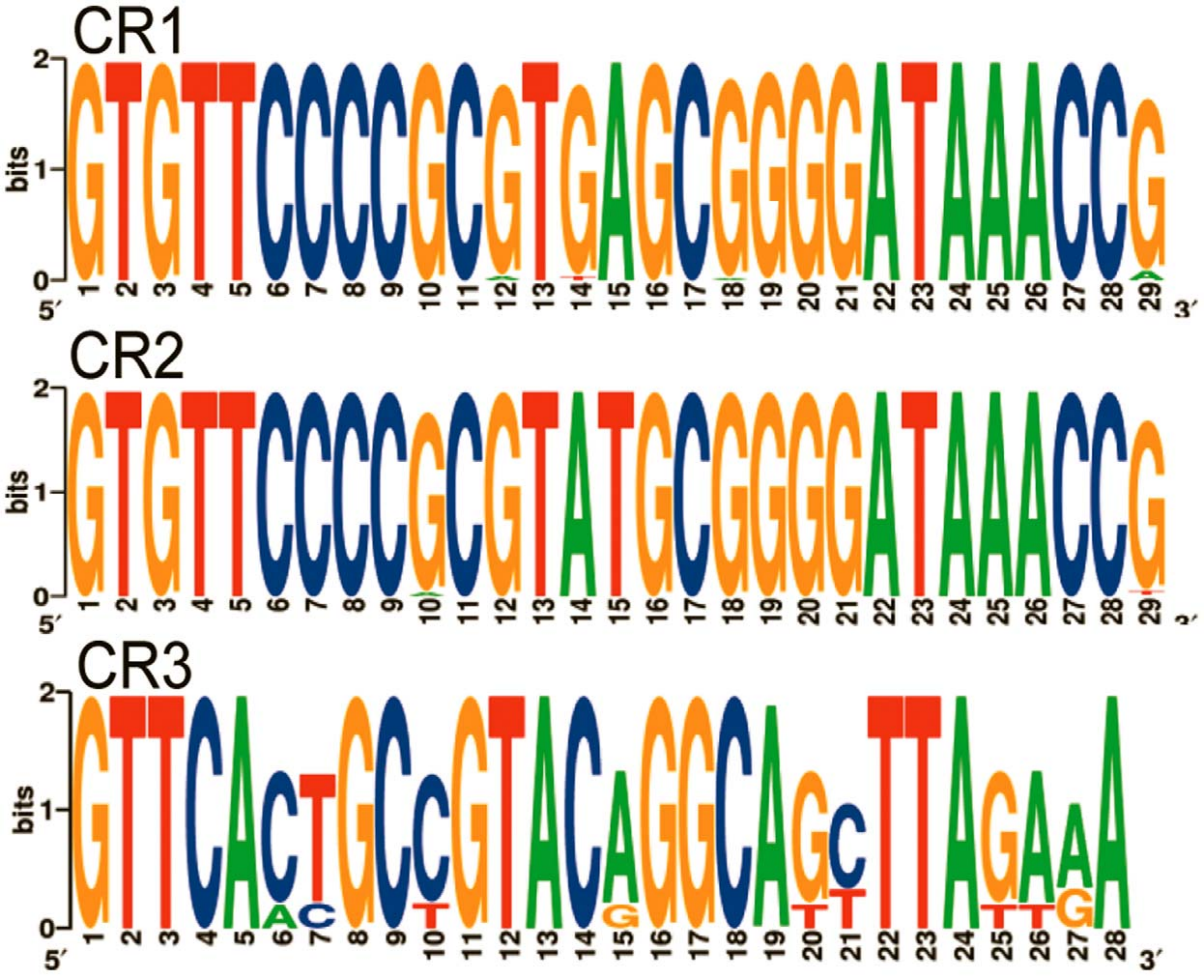
WebLogo representation of the sequence conservation of repeat sequences from CRISPR arrays CR1, CR2, and CR3 from *E. amylovora*. A total of 1630, 1979, and 345 repeats from CR1, CR2, and CR3, respectively, were utilized to generate the consensus sequences.

Cluster analysis was accomplished by the generation of an unweighted-pair group method (UPGMA)-based tree including all of the *E. amylovora* strains analyzed in this study. CRISPR spacers from the CRISPR 1, 2, and 3 arrays were concatenated and converted to a binary matrix based on presence or absence of a particular spacer sequence. The distance matrix was calculated using the Jaccard coefficient (http://genomes.urv.es/UPGMA/) with 1,000 bootstrap replications.

### Use of CRISPR Spacer Pattern in Strain Tracking

CRISPR spacer patterns were utilized to determine the identity of genotypes of *E. amylovora* in Michigan that were sensitive to streptomycin or that were identical to the three genotypes that had acquired the streptomycin resistance determinant Tn*5393* that carries the streptomycin resistance genes *strA* and *strB*. Similarly, we also examined the CRISPR genotype of *E. amylovora* S5, a natural spontaneous chromosomal Sm^R^ strain also isolated in Michigan. A total of 17 Sm^S^ *E. amylovora* strains (isolated between 1975 and 2011) and 24 Sm^R^ (Tn*5393*) *E. amylovora* strains (isolated between 1993 and 2011) were selected for this analysis.

### GenBank Accession Numbers

CRISPR array sequences were submitted to the NCBI database and assigned an accession number. A list of accession numbers by strain and array number (CR1, CR2, or CR3) is available in Table S2.

**Table 2:** Number of spacers located in CRISPR arrays 1 and 2 from *E. amylovora* strains isolated from apple and pear, *Rubus*, Indian Hawthorn, and loquat.

	Total spacers^a^		Minimum/Maximum spacer no.		Avg. no. of spacers ± SD	
	C1	C2	C1	C2	C1	C2
*E. amylovora* (85)						
Midwest, Eastern US and Canada (43)	36	34	27–36	23–34	34.7±3	26.3±4
Western US (16)	130	62	12–96	32–49	58.5±39	42.5±8
Europe and Middle East (11)	36	34	35–36	34	35.7±*1*	34*±*0
New Zealand (3)	35	34	35	34	35*±*0	34*±*0
Ea Rubus (6)	195	138	33–59	32–42	51.1*±*9	36*±*3
Ea Indian hawthorn (2)	29	42	29	42	29*±*0	42*±*0
Ea Loquat (4)	84	30	84	30	84*±*0	30*±*0

a The total count includes repeated spacers, if any.

## Results

### Global Genetic Organization of CRISPR Loci

Eighty-five strains of *Erwinia amylovora* varying in geographic isolation, plant host of isolation, plasmid content, and streptomycin sensitivity were evaluated for CRISPR array number and spacer variability (Table 1). Representative strains from Michigan, the eastern and western United States, Canada, Europe, the Middle East, and New Zealand were included in the study. Comparison of a 409-bp fragment of the *groEL* genes among strains revealed little polymorphism except for between strains from *Rubus* and the rest of the strains (Table 1).

**Figure 3:**
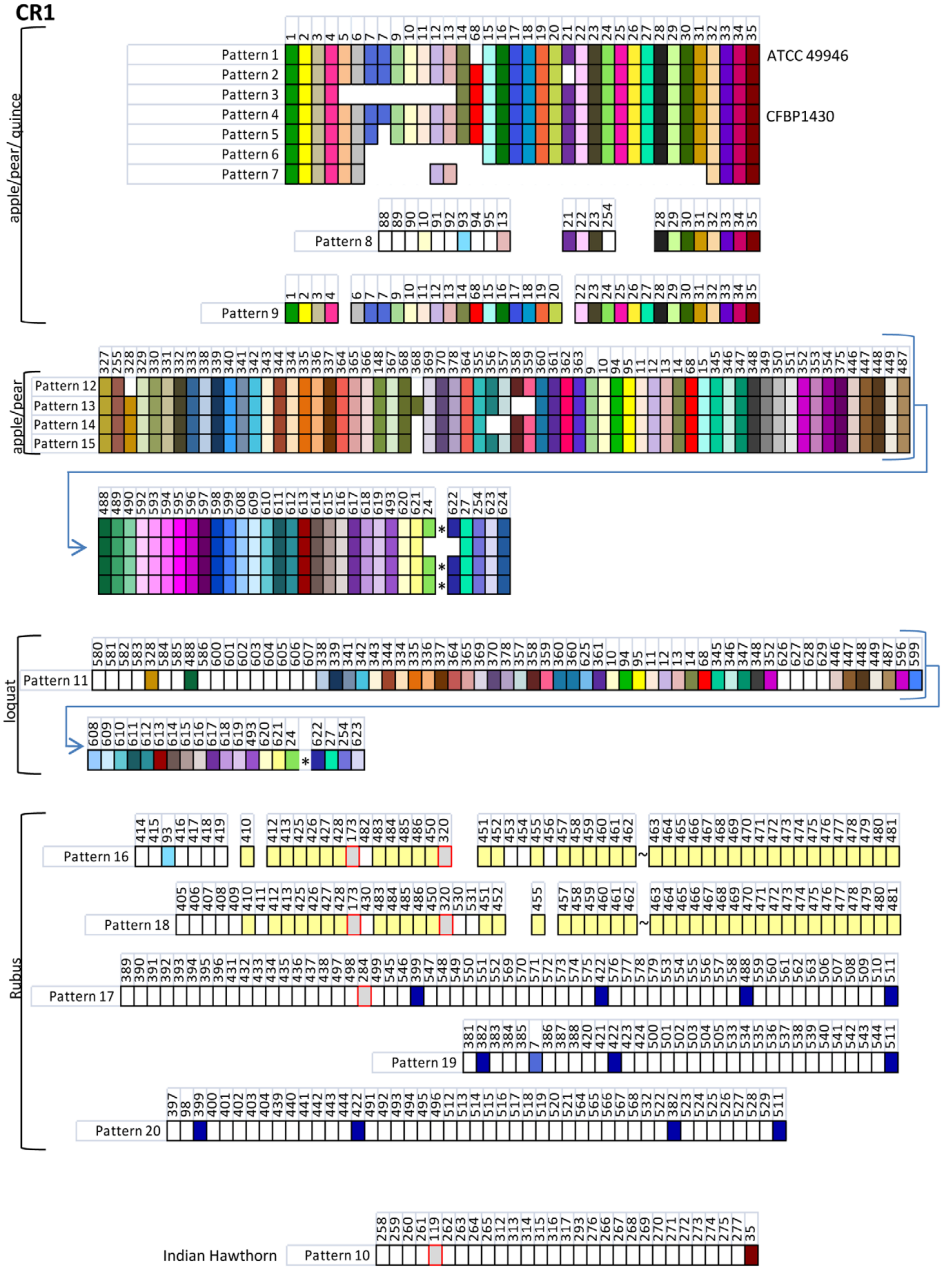
Graphic representation of spacers grouped into patterns from CRISPR array CR1 of the 85 *E. amylovora* strains examined in this study. Hosts of *E. amylovora* strains harboring the respective spacer patterns shown are listed on the left. Individual spacer sequences are represented by boxes; spacers were considered unique if they contained >5 nucleotide differences compared to other spacer sequences. Each unique spacer (588 identified in this study) was assigned a number. Spacers of similar color shown in the same columns under the same number are identical. Empty areas indicate the corresponding spacer in other similar patterns is not present. Open boxes refer to spacers that were only detected once in the collection. The “*” symbol refers to a 31-bp insertion sequence identified in 11 western U.S. strains from apple and pear and 4 loquat strains. The “∼” symbol refers to the location of a 10-bp insert (5′–gtgtgtgtgt–3′) observed in *E. amylovora* strains isolated from *Rubus*. Spacers shaded grey and boxed in red are spacers found in both CR1 and CR2 arrays, while spacers shared between *Rubus* strains are shaded in yellow or in dark blue. The following spacer patterns were predominantly isolated from *E. amylovora* strains from apple and pear isolated in the Midwest, eastern U.S., and Canada (CR1: patterns 1–4 and 9) and in the western U.S. (CR1: patterns 5–8 and 12–15).

We identified three arrays of spacer sequences associated with CRISPRs (CRISPR 1-3) present in *E. amylovora*. After our studies were initiated, genome sequences of two *E. amylovora* strains were completed, confirming the presence of three CRISPR spacer arrays in both sequenced strains [4], [5]. Arrays 1-3 are clustered together between nt 854678 to 879523 on the *E. amylovora* chromosome (nt position is relative to GenBank accession number NC_013971). The eight *cse* and *cas* genes are located between CRISPR spacer arrays 1 and 2 (Fig. 1) in an orientation that is termed the *E. coli*-type and is conserved in *Escherichia coli* [37], [38]. The 2.56-kb region between the 3′ end of the CRISPR array 1 and the *cas3* gene and the 9.46-kb region between CRISPR arrays 2 and 3 encoded *E. amylovora* housekeeping genes (Fig. 1).

**Figure 4:**
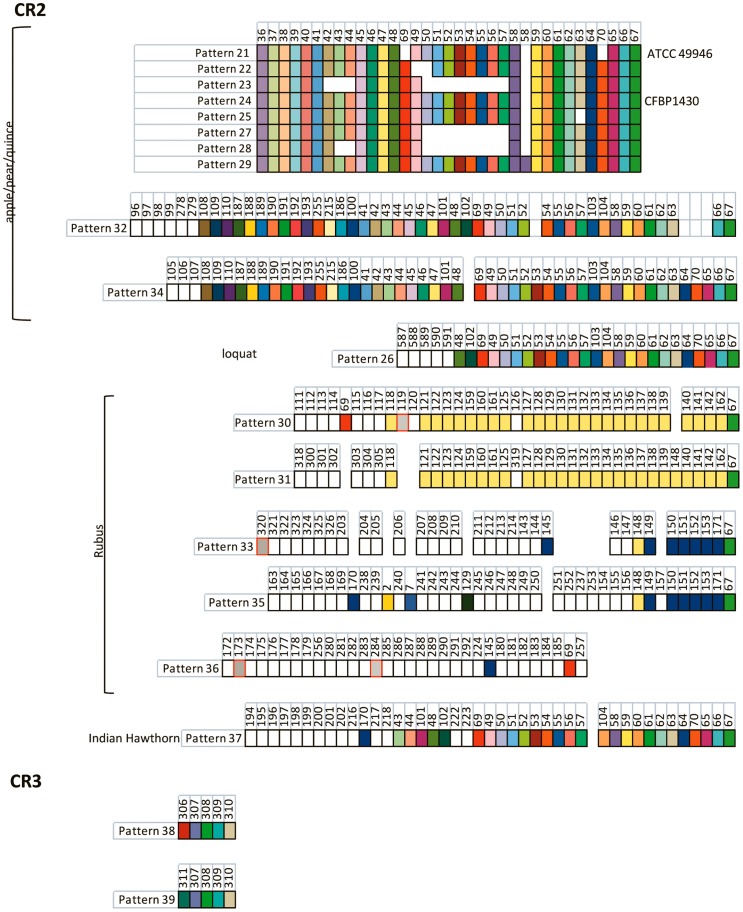
Graphic representation of spacers grouped into patterns from CRISPR arrays CR2 and CR3 of the 85 *E. amylovora* strains examined in this study. Hosts of *E. amylovora* strains harboring the respective spacer patterns shown are listed on the left. Individual spacer sequences are represented by boxes; spacers were considered unique if they contained >5 nucleotide differences compared to other spacer sequences. Each unique spacer (588 identified in this study) was assigned a number. Spacers of similar color shown in the same columns under the same number are identical. Empty areas indicate the corresponding spacer in other similar patterns is not present. Open boxes refer to spacers that were only detected once in the collection. Spacers shaded grey and boxed in red are spacers found in both CR1 and CR2 arrays, while spacers shared between *Rubus* strains are shaded in yellow or in dark blue. The following spacer patterns were predominantly isolated from *E. amylovora* strains from apple and pear isolated in the Midwest, eastern U.S., and Canada (CR2: patterns 21–28) and in the western U.S. (CR2: patterns 29, 32, and 34).

The genetic organization of the *cse* and *cas* genes found in the necrogenic *Erwinia* spp., i.e. *E. amylovora*, *E. pyrifoliae*, and *Erwinia* sp. from Japan, is identical and the translated Cse and Cas proteins share high levels of amino acid identity (95% and 92% identities of Cas1 from Ejp617 and Ep1/96, respectively; 89% identities of Cas3 from Ejp617 and Ep1/96; 92% identities of Cse1 from Ejp617 and Ep1/96). In contrast, the gene organization in *E. tasmaniensis*, a related non-pathogenic bacterium from apple and pear, most closely resembles the gene organization termed the Ypest-type that is present in *Yersinia pestis* [38], [39]. Likewise, the *cse* and *cas* gene organization in *Pectobacterium atrosepticum* (GenBank accession no. BX950851.1), a soft rot pathogen related to *E. amylovora*, was also the Ypest-type. Interestingly, *Dickeya dadantii* (GenBank accession no. NC_004547), another related soft rot pathogen of vegetable and ornamental crops, possesses two sets of CRISPR associated genes, one each of the Ecoli- and Ypest-types.

**Table 3:** CRISPR array genotype distribution by host and by location of strain isolation.

	Number of Ea isolates found with pattern		
CRISPR array PatternCR1-CR2-CR3	Midwest/Eastern U.S./Canada	Western U.S.	Europe/Middle East/New Zealand
**Apple/pear/quince**			
1-21-38	1	0	0
2-22-38	4	0	0
3-23-38	1	0	0
3-24-38	3	0	0
4-22-38	2	1	0
4-23-38	21	0	0
4-24-38	1	0	8
4-25-38	1	0	0
4-27-38	4	0	0
4-28-38	1	0	0
5-24-38	0	2	6
5-27-38	1	0	0
6-24-38	1	0	0
7-24-38	0	1	0
7-29-38	0	3	0
8-32-38	0	1	0
9-23-38	2	0	0
12-34-38	0	3	0
13-34-38	0	1	0
14-34-38	0	1	0
15-34-38	0	3	0
**Total no. of patterns by region**	**13**	**9**	**2**
**Indian Hawthorn**			
10-37-38	2	na	na
**Loquat**			
11-26-38	4	na	na
***Rubus***			
16-30-39	2	na	na
17-36-39	1	na	na
18-31-39	1	na	na
19-35-39	1	na	na
20-33-39	1	na	na

### Characterization of CRISPR Repeat Sequences within *E. amylovora* and Related Species

The CRISPR 1 and 2 repeat sequence found in all *E. amylovora* strains in this study consisted of 29 bp and was universal despite host range or other variables (Fig. 2). Two nucleotide substitutions (GA to AT) at positions 14 and 15 were identified that differentiated CRISPR 1 and CRISPR 2. *Erwinia pyrifoliae* is the only organism in the CRT Data base (http://crispr.u-psud.fr/crispr/) with repeat sequences that share 100% sequence identity to the CRISPR 1 and 2 repeats found in *E. amylovora*. While there were no other organisms with CRISPR repeats identical to those found in *E. amylovora* with the exception of *E. pyrifoliae*, a comparison to closely related species in the CRT database revealed similarity to CRISPR repeats found in other enterobacteria including *Enterobacter sakazakii*, *E. coli*, *Citrobacter rodentium*, *Salmonella enterica* subsp. *enteric*a, and *S. typhimurium*. The 28 bp repeat for array CR3 was distinct from those found in arrays CR1 and CR2 (Fig. 2). Other organisms with array repeats identical to CR3 are *Shigella flexneri* 2a str. 301, *Pectobacterium atrosepticum* SCRI1043, *Photorhabdus luminescens* subsp. *laumondii* TTO1, *Legionella pneumophila* str. Lens, *Shigella sonnei* Ss046, and multiple *E. coli* isolates.

**Figure 5:**
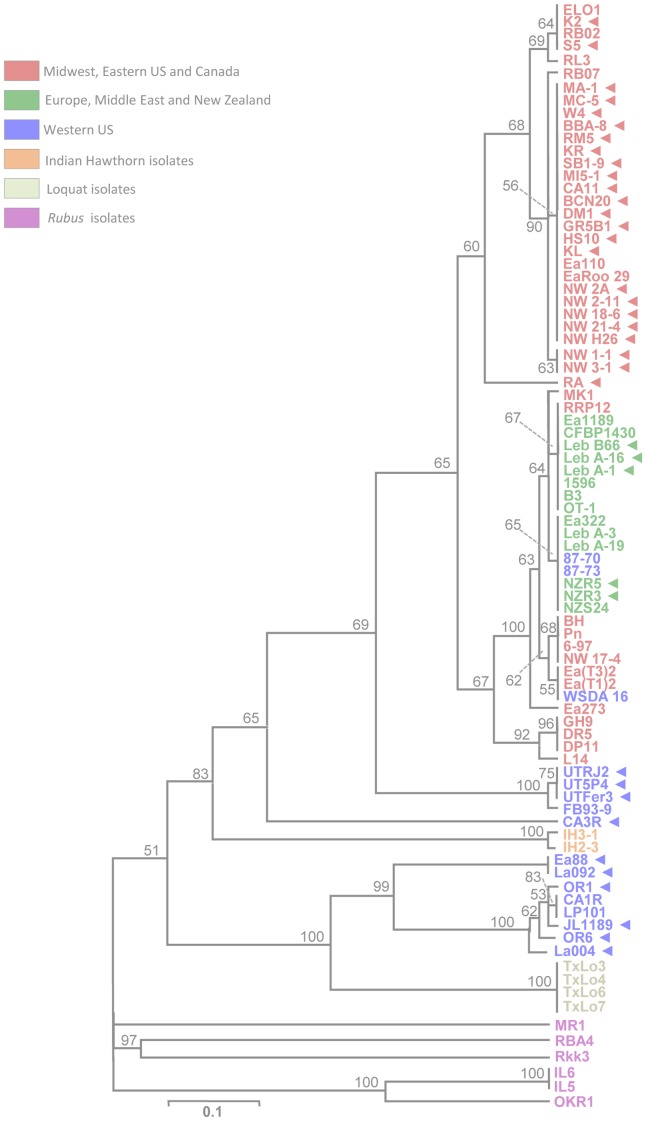
Cluster analysis of concatenated spacer patterns from CRISPR arrays CR1, CR2, and CR3 of 85 *E. amylovora* strains. Strains isolated from apple and pear are distinguished by geographic origin and shown in red (midwestern and eastern U.S. and Canada), blue (Western U.S.), or green (Europe, Middle East, and New Zealand). Strains from other hosts are shown in orange (Indian hawthorn), light green (loquat), and magenta (*Rubus*). Triangles to the right of strain names delineate streptomycin-resistant strains. Bootstrap values >50% are shown.

### Sequence Analysis and Examination of CRISPR Spacer Repertoire

A total of 588 individual spacers were identified among the 85 strains analyzed in this study within the three CRISPR arrays present in *E. amylovora* (Table S3). A spacer was considered unique if it contained >5 nt changes relative to other spacers. Each unique spacer was assigned a number that was used to aid in assembly and comparison of patterns. Spacers with five or fewer differences were considered “variants” of previously characterized spacers and assigned the same spacer number in the spacer catalog. Spacers were characteristically 32 bp in length. The number of spacers contained within spacer arrays CR1 and CR2 was variable among strains and ranged from 12 to 96 and 23 to 49 within arrays CR1 and CR2, respectively. All strains contained 5 spacers in array 3.

**Figure 6:**
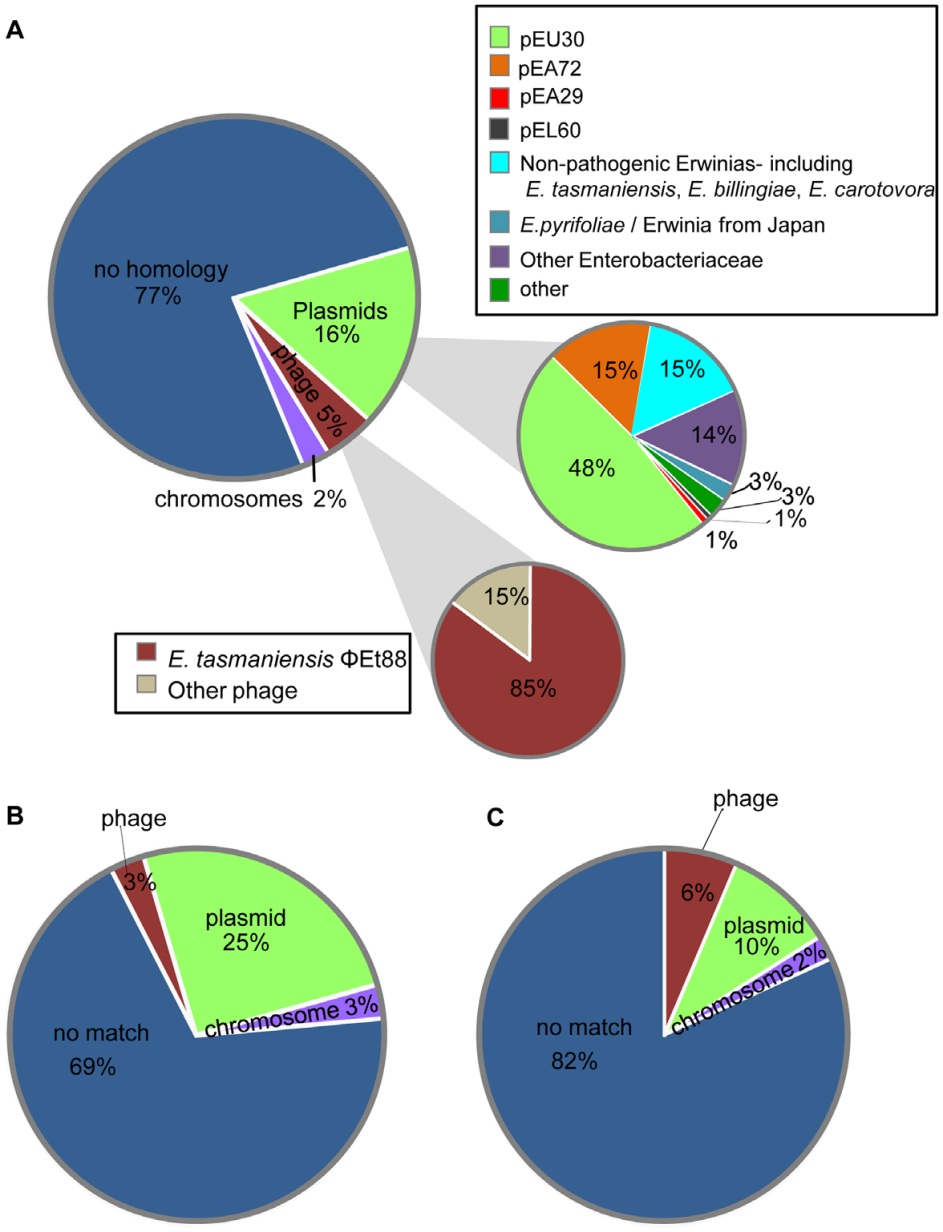
CRISPR spacer distribution in plasmids and bacteriophages determined by sequence identity. Percent distribution of CRISPR spacers from (A) all 85 *E. amylovora* strains examined in this study, (B) 73 *E. amylovora* strains isolated from apple or pear, and (C) 6 *E. amylovora* strains isolated from *Rubus* sharing sequence identity with plasmids, bacteriophage, and chromosomal sequences as well as percentage of spacers with no known homology to sequences in the GenBank database. Spacers from all *E. amylovora* strains shown in (A) with plasmid homology are further subdivided into percentages with homology to pEU30, pEA72, pEL60, and pEA29 from *E. amylovora*, plasmids from *E. pyrifoliae* and *Erwinia* sp. pathogens, plasmids from non-pathogenic *Erwinia* spp., plasmids from other Enterobacteriaceae, and other plasmids. Spacers from *E. amylovora* with bacteriophage homology are further subdivided into percentages with homology to *E. tasmaniensis* phage ΦEt88 and to other phage sequences. A total of 14 spacers with identity to plasmid sequences matched >1 plasmid. These s were counted a single time when placed into group homologies (i.e., plasmid, phage, and chromosomal) and multiple times as necessary for plasmid characterizations.

For analysis purposes, the *E. amylovora* strain collection utilized for study was differentiated by geographic location of isolation and also by host of isolation [apple (*Malus* x *domestica*) and pear (*Pyrus communis*), *Rubus* sp., Indian hawthorn (*Rhaphiolepis indica*), and loquat (*Eriobotrya japonica*)]. The western U.S. and *Rubus* groups, along with the strains isolated from loquat, contained an increased number of spacers within the CRISPR 1 (CR1) array with a high of 96 spacers in strains Ea88, La092, and JL1189 from Washington (Table 2). Another biological aspect in addition to number of spacers is the similarity and uniqueness of spacers present in particular strain groups. Most strains isolated in the eastern U.S. and Canada contained similar spacers in similar order in CR1 and CR2 arrays (Fig. 3 and 4). CR1 and CR2 arrays from western U.S. strains contained some of these spacers, but many were distinct, although conserved in the western group (Fig. 3 and 4). The six strains from *Rubus* displayed five different CR1 and CR2 arrays, only two of which were somewhat similar. Most of the spacers in the *Rubus* strains were unique to the entire collection (Fig. 3 and 4). The loquat strains all shared the same spacers, and both the CR1 and CR2 arrays in these strains were closely similar to western U.S. apple and pear strains (Fig. 3 and 4). A total of 79% of the spacers from the CR1 and CR2 patterns of the loquat strains was shared between the loquat and western U.S. apple and pear strains.

**Figure 7:**
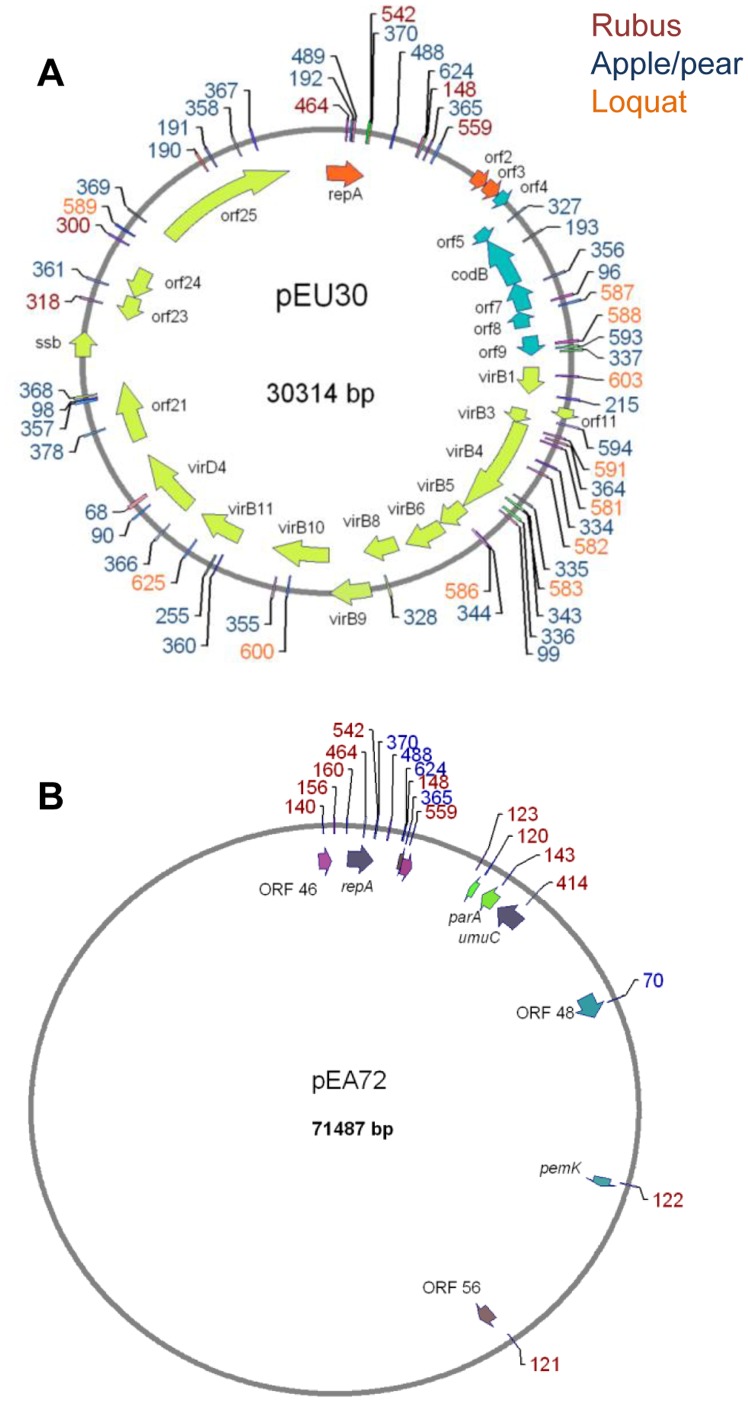
Target locations of CRISPR spacer sequences from *E. amylovora* strains in plasmids pEU30 and pEA72. Spacers are numbered as in Fig. 3 and shown in blue, maroon, and orange based on their presence in CRISPR arrays from *E. amylovora* strains isolated from apple and pear, *Rubus*, and loquat, respectively. The annotated gene map showing the open reading frames of pEU30 is included, from [20]. A partial annotation of pEA72 is shown as relevant to target locations in this plasmid.

**Figure 8:**
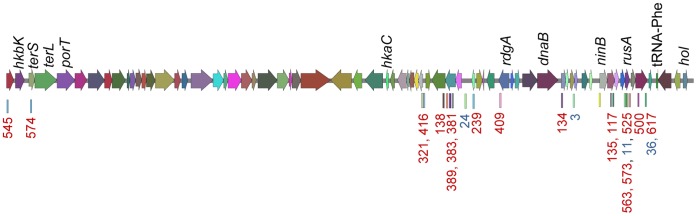
Target locations of CRISPR spacer sequences from *E. amylovora* strains in bacteriophage ΦEt88 from *E. tasmaniensis*. The annotated gene map, showing the open reading frames of ΦEt88, was constructed using the sequence available in GenBank (Accession number FQ482085).

In addition, the western U.S. strains contained 122 spacers in arrays 1 and 2 that were not identified in other apple or pear strains from other geographic locations. In contrast, each spacer present in strains isolated from the eastern and midwestern U.S. was also found in at least one other strain from a different geographic source or different host. Host of isolation was also important as the *E. amylovora* strains from *Rubus*, Indian hawthorn, and loquat exhibited completely different spacer arrays or array patterns, and most of the spacers found were unique to those strain groups. The diversity among spacers in the *Rubus* strains was quite extensive, accounting for 320 unique spacer sequences, compared to 54, 41, and 24 unique spacers in apple/pear, Indian hawthorn, and loquat strains, respectively.

Alignment of spacer sequence patterns from each CRISPR array indicated the presence of distinct genotypes among the strains examined. Each pattern was given a numerical designation (Fig. 3) with a total of 20, 17, and 2 patterns identified among spacer arrays CR1, CR2, and CR3, respectively (Fig. 3 and 4). Groups of similar patterns within a single array could be distinguished by the deletion of individual spacers or by deletion of contiguous groups of spacers (for ex., deletion of spacers 5–13 in CR1, pattern 3; Fig. 3). Single spacers or small blocks of spacers were also located in arrays that were otherwise completely different (for ex., presence of spacers 9, 10, 94, 95, 11–14, 68 in CR1, patterns 11–16; Fig. 3).

**Table 4:** CRISPR array genotype for CRISPR arrays CR1, CR2, and CR3 in streptomycin sensitive and Sm^R^ *E. amylovora* strains isolated from apple in Michigan. Patterns listed in bold for streptomycin-sensitive strains were also observed in Sm^R^ strains.

CR1-CR2-CR3 Pattern	No. of Sm^S^ strains isolated in Michigan	No of Sm^R^ strains isolated in Michigan
2-22-38	3	0
3-23-38	0	1
3-24-38	3	0
4-22-38	2	0
**4-23-38**	1	20
4-24-38	1	0
4-25-38	1	0
**4-27-38**	**3**	**1**
4-28-38	1	0
5-27-38	1	0
6-24-38	1	0
9-23-38	0	2

In 15 *E. amylovora* strains from the western U.S. (11/16 strains) and loquat (4/4 strains), 31 bp of sequence interrupting the CR1 array was identified inserted between spacer 24 and the repeat sequence preceding spacer 622 (Fig. 3 patterns 11, 12, 14, and 15). This insert (5′ – gtgttcccgctcttttgcggcttagtgcgaa- 3′) lacked homology to the repeats identified in *E. amylovora* or to other CRISPRs when compared to the CRISPR repeat database (http://crispr.u-psud.fr/crispr/BLAST/CRISPRsBlast.php). Searches in both the CRISPR spacer database and to GenBank by BLASTn revealed no significant matches greater than 18 nt. This insert was not observed in CR1 pattern 13 (Fig. 3) which may have undergone a homologous recombination event that deleted this insert and the flanking spacers. A second novel insert was detected in CR1 arrays 16 and 18 from 3 *E. amylovora Rubus* strains. This 10-bp insert consisted of 5 repeating GT pairs and was located between spacer 462 and 463 downstream of the 462 spacer.

Five *E. amylovora* strains (Ea110, Ea(T1)2, Ea(T3)2, EaRoo29, and *Rubus* isolate RKK3) with known differing profiles of bacteriophage sensitivity to ΦEa1, ΦEa7, ΦEa100, ΦEa125, and ΦEa116C were included in this study [29 and unpublished data]. Three CRISPR genotypes were identified in the 5 strains. Although their phage sensitivity differed, CRISPR genotype 4-23-38 was shared between Ea110 and EaRoo29, and genotype 4-22-38 was shared between isolates Ea(T1)2 and Ea(T3)2.

The grouping of strains based on combined patterns for CRISPR spacer arrays 1, 2, and 3 resulted in the identification of 28 distinct genotypes with clear differentiation by host of isolation and geographic location (Table 3). Similarity of genotypes among *E. amylovora* strains isolated from apple and pear in the eastern U.S. was the most readily-apparent feature identified. However, we observed an almost complete lack of overlap (only one exception) of CRISPR genotype among apple and pear strains isolated from the eastern or western U.S. (Table 3). Of two CRISPR genotypes identified among apple and pear strains isolated in Europe, the Middle East, or New Zealand, one each was identified in a strain isolated in the eastern or western U.S. (Table 3). The genotypes of strains isolated from *Rubus*, Indian hawthorn, or loquat were all unique when compared to the apple and pear strains due to the presence of additional unique spacers (Table 3). A complete listing of the combined CRISPR spacer array pattern for each of the 85 strains analyzed in this study is presented in Table S4.

Cluster analysis of the concatenated CRISPR sequence arrays led to the differentiation of genotypic groups that further highlighted the similarities and differences among strains related to geographic source and host of isolation (Fig. 5). Of particular interest was the separation of most of the apple and pear strains by geographical location of isolation, and the overall similarity in arrays of loquat and western U.S. apple and pear strains (Fig. 5). It should be noted that branch lengths in the tree are biased by the effect of size of the CRISPR array regions. Thus, the clustering and branching identified is not indicative of *E. amylovora* phylogeny but is likely caused by rapid divergence or replacement of CRISPR arrays within individual strains.

### CRISPR Spacer Repertoire from *E. pyrifoliae* and *Erwinia* sp

Although *E. amylovora* and *E. pyrifoliae* shared 100% sequence identity for CR1 and CR2 repeats, they did not share spacer homology. Examination of *E. pyrifoliae* CRISPR genotypes revealed conservation of a single genotype among strains Ep16/96, Ep1/96 and Ep4/97 with spacer organization and sequence conserved (data not shown) suggesting that the limited number of isolates available to us for use in this study were closely related to one another. Conversely, examination of the CRISPR profiles from 3 strains of *Erwinia* sp. isolated from Japan thought to be closely related to both *E. amylovora* and *E. pyrifoliae*, revealed conserved repeat sequences among strains but no single conserved CRISPR genotype and a diverse spacer repertoire. At the time experiments were conducted, there were no genome sequences available for Japanese *Erwinia*. Despite repeated attempts, we were unable to amplify the CR1 array from Ejp556 or Ejp557 using primers EPyF-1 and EPyR-1 or any of the *E. amylovora* CR1 primers listed (Table S1); however, amplification of the CR2 and CR3 arrays from Japanese *Erwinia* using primers designed from *E. pyrifoliae* sequences was successful. With the genome of Japanese *Erwinia* sp. strain Ejp617 (GenBank accession CP002124) now available [15], the sequence of the CRISPR arrays from Ejp617 are known. Spacers from Japanese *Erwinia* CR1 (Ejp617 only) 2 and 3 arrays shared no homology to spacers from *E. amylovora*. A single spacer, designated Ep3, was shared between *E. pyrifoliae* CR1 and Ejp617 CR2 but was not found Ejp556 or Ejp557 CR2 and CR3 arrays. A diagrammatic representation of the spacer arrays from *E. pyrifoliae* and *Erwinia* sp. strains is shown in Fig. S1.

### Relationship of CRISPR Spacer Sequences with known Plasmid and Bacteriophage Sequences

The sequence identity of the 588 individual CRISPR spacers identified in this study with other known sequences was determined using BLASTn analysis, and a total of 77% of the spacers had no match to the GenBank databases. The remaining 23% of spacers examined shared nucleotide identity with plasmids (16%), bacteriophage (5%) or bacterial chromosomal DNA sequences (2%) (Fig. 6). We defined positive relationships as the sharing of sequence identity among at least 25 of 32 nucleotides (>78%) in a given spacer with an existing sequence(s) in the GenBank database.

Almost all of the 95 spacers matching known plasmid sequences targeted plasmids previously identified in *E. amylovora*, other *Erwinia* spp., or other Enterobacteriaceae (Fig. 6), with a disproportionate bias toward pEU30 (targeted by 55 spacers), a conjugative plasmid only present in *E. amylovora* strains isolated from the western U.S. [20]. *E. amylovora* strains that were isolated from apple, loquat, and *Rubus* hosts possessed spacers with homology to pEU30. Forty-four of the 51 spacers shared 100% sequence identity with the pEU30 sequence from *E. amylovora* UTRJ2 with an apparent unbiased distribution of targets within the plasmid (Fig. 7A). The majority of the spacers with homology to pEU30 were present in the CR1 array; only 6 of 55 pEU30-spacers were located in CR2 arrays. A total of 5 of 16 of the *E. amylovora* strains from the western United States analyzed in this study harbored pEU30. Two of these five strains (UTRJ2 and UT5P4) were isolated in Utah and had no spacers matching pEU30. However, the remaining three strains (JL1189, LA092, and La004), isolated from pear, harbored pEU30, but also contained numerous spacers that matched the pEU30 sequence. Any *Rubus* or loquat strains of *E. amylovora* with spacers matching pEU30 did not harbor the plasmid. In addition, none of the spacers with homology to pEU30 from *Rubus* or loquat strains were shared with the three pear strains from the western U.S.

A single spacer (no. 214 in this study; Table S3) from *Rubus* strain Rkk3 shared 100% identity (32/32 bp) with the *repA* gene of pEA29, the ubiquitous plasmid found in *E. amylovora* [18]. Other significant nucleotide matches of spacers to plasmids in the necrotizing *Erwinia* group included *E. amylovora* pEA72, a 71.4 kb plasmid found in Ea273/ATCC 49946 (NC_013971) (Fig. 7B), pEL60 from *E. amylovora* strain Leb B66 [20], pEP36 from *E. pyrifoliae* [22], pET35 and pET46 from *E. tasmaniensis* [40], pEB170 from *Erwinia billingiae* [40], pEJ30 and pJE02 from *Erwinia* sp. from Japan [12], [15], and pEAR4.3 from *E. amylovora Rubus* strain IL-5 [13]. Unlike with pEU30, most spacers targeting *E. amylovora* pEA72 targeted sequences located close to the replication origin of the plasmid (Fig. 7B).

*E. amylovora* strain Ea273 ( =  ATCC 49946) was the only strain examined from the midwest/eastern U.S./Canada group with a CRISPR array lacking the spacer with homology to *E. amylovora* pEA72 (no. 70; Table S3). Ea273 is also the only strain examined in the study possessing *E. amylovora* pEA72 (data not shown). Significant spacer similarity was also observed with plasmids isolated from bacterial species in the *Enterobacteriaceae* family, including plasmids from *Salmonella enterica* subsp. enterica, *Yersinia pestis*, *Yersinia pseutotuberculosis*, and *Pectobacterium carotovorum* subsp. *carotovorum* (data not shown).

Of the 26 spacers identified with identity to bacteriophage sequences, 22 were located within *E. tasmaniensis* bacteriophage ΦEt88 (Fig. 8). Four of the 22 spacers were found in strains from apple and pear, 1 of the spacers was found in strains from apple and pear and loquat, and the remaining 17 were identified in *E. amylovora* strains recovered from *Rubus*. The only other notable match to bacteriophage was found in spacer no. 449 (Table S3) isolated from western strains of *E. amylovora*. This spacer had similarity (28/29 bp) to *Burkholderia* bacteriophage ΦE12-2 to the coding region for a bacteriophage capsid scaffolding protein (data not shown). Sequences for *E. amylovora* bacteriophage ΦEa100 and ΦEa1 are available [30]. Comparison of *E. amylovora* isolates reported to be resistant to one or both of these bacteriophage had no spacer homology to either phage.

Typically, CRISPR spacers with homology to chromosomal DNA targeted sequences that were similar to bacteriophage integrases, a topoisomerase gene, and a *pilV* gene (data not shown).

### Use of CRISPR Spacer Patterns for Tracking the Dispersal of Sm^R^ *E. amylovora* in Michigan

CRISPR genotypes of Sm^R^ and Sm^S^ *E. amylovora* strains isolated in Michigan were compared. The Sm^S^ strains, isolated early as 1975, exhibited diverse CRISPR spacer pattern profiles among CRISPR arrays 1, and 2, with 10 genotypes observed among 17 strains (Table 4). In contrast, 20 of 24 Sm^R^ strains from Michigan (first isolated in 1993) exhibited an identical CRISPR genotype (genotype 4-23-38, Table 3). The correlation of CRISPR genotype with streptomycin sensitivity or resistance status resulted in the identification of potential Sm^S^ ancestral strains for 21 of the 24 Tn*5393*-containing Sm^R^ strains studied (Table 4). In addition, the CRISPR genotype of the spontaneously Sm^R^ strain S5, containing a mutation in the *rpsL* gene [25], [27] was also observed in three Sm^S^ Michigan genotypes (Table 4).

## Discussion

Analysis of CRISPR spacer sequences and patterns revealed considerably more genetic diversity in *E. amylovora* than had been known previously. CRISPR genotyping enabled the differentiation of strains that were shown in previous studies to be contained within the same ribotype, pulsed-field gel electrophoresis (PFGE) group, and *groEL* sequence group [6], [8], [27]. *E. amylovora* has been considered a fairly homogeneous species with a low level of genetic diversity although there are obvious differences between genomes of strains isolated from apple and strains isolated from *Rubus* sp. [13]. This is due to the hypothesis of a recent evolutionary bottleneck associated with the colonization of apple hosts in North America in the 1700s. The previous host(s) of the progenitor strain(s) first infecting apple and pear is unknown, and, to our knowledge, a comprehensive phylogenetic analysis of *E. amylovora* including a large number of strains isolated in North America from wild Rosaceae hosts has not been done. While the CRISPR locus is not useful for phylogenetic analyses [41], we and others [42] have shown that CRISPR spacer array genotyping is a potential tool that could be used to identify progenitor strain(s) of *E. amylovora* that are currently infecting apple and pear today. In this study, the *E. amylovora* strains isolated from loquat were closest in genetic relatedness of CRISPR spacer content with apple and pear strains.

CRISPR spacer sequences are thought to provide a historical context of mobile sequences an organism encounters because individual spacers are inserted at the same position, adding on to the existing spacer assembly [36]. For most of its life cycle, *E. amylovora* is believed to be present within the interior of plants with significant epiphytic growth only occurring on the stigma surfaces of flowers [2]. However, the recent identification of *E. amylovora* pathogenicity island sequences suggestive of functioning in association with insect hosts has broadened the habitats in which this bacterium may dwell [43]. Thus, the diversity of *E. amylovora* habitats (plant and insect) could potentially increases the ecological context of CRISPR spacer evolution with exposure to distinct microbiomes.

Fire blight disease and *E. amylovora* were known to spread from North America to New Zealand in the 1910s, to Europe in the 1950s, and subsequently to the Middle East [44], [45]. Our results of strain genotyping based on CRISPR spacer content lead us to hypothesize that an *E. amylovora* strain(s) from the eastern U.S. is the likely source of fire blight disease spread into New Zealand and Europe. This is due to the observation that the most prevalent eastern U.S. genotype only differs from the genotypes observed in European and New Zealand strains by a small set of deleted spacers (Table 3 and Fig. 3). Spacer deletion is thought to be a common route to CRISPR genotypic differentiation [46]. Based on previous work using PFGE analyses, Jock and Geider [45] concluded that *E. amylovora* was originally spread from North America to England, and from there likely throughout Europe. PFGE results also suggested that *E. amylovora* was not repeatedly introduced from North America, rather only a few strains, in effect a bottleneck were associated with spread of *E. amylovora* to Europe [6]. Our CRISPR spacer data, and those of a previous study [42], corroborate these results, as relatively little diversity is observed among European *E. amylovora* strains while North American strains contain a higher level of diversity.

Our current results also suggest that the sources of *E. amylovora* strains initially infecting apple and pear in the eastern and western U.S. could be distinct. *E. amylovora* WSDA 16, 87–70, and 87–73 are the only western U.S. strains with similar CRISPR genotype to eastern U.S. strains (Fig. 5). It seems likely that these strains were transported from the eastern U.S. to the western U.S. through human activity such as via movement of contaminated nursery stock.

We observed a clear delineation in spacer content and diversity in the CRISPR 1 and 2 arrays in the *E. amylovora* strains studied, based on geographical location of isolation and plant host. This differentiation of spacer content lends credence to the hypothesis that CRISPR spacers reflect a geographic component of host interactions and an environmental niche component [32], [36], [39]. Geographic differentiation within CRISPR loci has been used previously to reconstruct the routes of transmission of *Yersinia pestis* strains from natural plague foci in China [47] and to make inferences about viral biogeography, host-virus interactions, and genome dynamics in *Sulfolobus islandicus* [48]. For the most part, *E. amylovora* apple strains from the western U.S. harbored a completely distinct set of CRISPR spacers compared to corresponding strains from the eastern U.S. and other continents, and many of the spacers carried by the western strains targeted pEU30, a plasmid that is exclusively found in a subset of western U.S. apple strains [20].

Differentiation of CRISPR spacer content based on host of isolation adds to the possibility of an environmental niche component affecting the evolution of CRISPR loci. The *E. amylovora* strains isolated from *Rubus*, are readily differentiable from apple strains by various typing methods, and this has been confirmed at the genome sequence level [13]. These strains are also differentiated by host specificity in that *Rubus* strains are not pathogenic on apple or pear [49], [50]. We found that these strains are also clearly distinct in terms of CRISPR genotype. Our results, along with the clear phylogenetic distinction of *E. amylovora* strains isolated from apple and from *Rubus* [8], [12], suggests that these strains have been evolving in isolation from each other for an evolutionarily long period of time. Our results are also similar to those observed in *E. coli*: when phylogenetic distance is small, a high relatedness of spacer repertoire is observed [41]. As phylogenetic distance increases, spacer relatedness decreases. However, a second aspect of the *E. coli* analyses indicated that spacer repertoire relatedness among strains followed either of two paths: the spacer content was either closely identical or completely different [41]. This radical replacement or replenishment of spacers with unique spacers was interpreted to indicate that turnover of spacers is not gradual [41]. Our results with *E. amylovora* corroborate these previous observations with *E. coli*.

The utility of CRISPR sequences for strain tracking on a local level was demonstrated in this study as we detected similar CRISPR genotypes in Michigan populations of Sm^S^ *E. amylovora* and in corresponding Sm^R^ strains that had either acquired Tn*5393* or were spontaneous Sm^R^ mutants. Thus, CRISPR analysis was more sensitive than comparative *groEL* sequencing or ribotyping which were used previously in an attempt to differentiate these strains [3], [27]. These results are important in that they suggest that Sm^R^ *E. amylovora* populations in Michigan evolved from indigenous populations which also suggests that the resistance has arisen in locally-adapted genotypic backgrounds.

The diversity and distribution of plasmid sequences inhabiting *E. amylovora* has received increased attention in recent years as researchers attempt to define the pan-genome of this species [21], [24], [51], [52]. Identification of 95 spacers targeting plasmids found in *Erwinia* spp. in this study provides evidence of prior interactions and attempts to avoid incursions of specific plasmids during the life history of these strains. Of particular interest are spacers targeting plasmids reported from the epiphytic organisms *E. billingiae* and *E. tasmaniensis* and other related pathogenic species *E. pyrifoliae* and *Erwinia* sp. isolated in Japan (Fig. 6). Our observations either suggest interactions of *E. amylovora* with these other species or mobility of targeted plasmids into *E. amylovora* at points during the life history of this pathogen. Another question originating from our analyses is why is pEU30 targeted by so many spacers? Analysis of the complete sequence of pEU30 [20] suggested that the plasmid is relatively innocuous; aside from a *virB*-type system encoding conjugation machinery, the plasmid does not encode any known genes of ecological or pathogenic importance. The lack of traits encoding a positive fitness benefit might be the very reason that pEU30 is frequently targeted for elimination. In addition, we found that three strains that harbored pEU30 also contained CRISPR spacers targeting the plasmid. Since it is known that 100% nucleotide identity is required for sequence elimination by the CRISPR system [34], this could be an example of a plasmid-bacterial host “arms race” in which the plasmid has evolved through mutation to escape CRISPR surveillance. An alternate hypothesis is that self-targeting CRISPRs are involved in gene regulation; however, a recent comprehensive analysis suggested that self targeting is more a consequence of autoimmunity [53].

In a recent study with *E. coli* in which 926 unique spacer sequences were identified, none of these were found to match any known sequenced enterophages [39]. This discontinuity between CRISPR sequences and bacteriophage sequences could be due to the low availability of phage sequences compared to phage environmental diversity. We identified 22 spacers targeting known phage sequences, and most of these targeted phage ΦEt88, which was previously identified in *E. tasmaniensis* [14]. Our results could also be due to the low availability of phage sequences or to the lack of encounters between the *E. amylovora* examined in this study and these characterized phages. The potential of phage deployment for fire blight disease management has been assessed by several groups [28], [30], [54], [55]. Since the sensitivity or resistance to infection by specific phage can be affected by genes in addition to the CRISPR loci, much more information would be necessary to predict the sensitivity of *E. amylovora* strains to phage under development for fire blight control.

In summary, we characterized CRISPR spacer diversity among 85 *E. amylovora* strains and found that this locus is robust for differentiating genotypes. We find that CRISPR analysis could be particularly useful for strain tracking on a local and possibly on a regional level. Also, the almost completely distinct composition of CRISPR arrays between *E. amylovora* strains isolated in the eastern and western U.S. indicates the potential that there were multiple introductions of this pathogen from native Rosaceae hosts to apple and pear hosts brought to and transported across North America by European settlers.

## Supporting Information

Figure S1 **Graphic representation of spacers grouped into patterns from CRISPR arrays CR1, CR2, and CR3 of** ***E. pyrifoliae*** **and** ***Erwinia*** **sp. strains.** Individual spacer sequences are represented by boxes; spacers were considered unique if they contained >5 nucleotide differences compared to other spacer sequences. Each unique spacer was assigned a number with an Ep (*E. pyrifoliae*) or EJP (*Erwinia* sp.) prefix. Only spacer Ep3 was shared among *E. pyrifoliae* and *Erwinia* sp. (strain 617). Empty areas indicate the corresponding spacer in other similar patterns is not present. ND indicates that the sequence of CR1 was not determined for *Erwinia* sp. 556 and 557.
(TIF)

Table S1 **Oligonucleotide primers used in this study and their description.**
(DOCX)

Table S2 **GenBank accession numbers of CRISPR spacer array sequences CR1, CR2, and CR3 for each** ***E. amylovora*** **strain determined in this study.**
(DOCX)

Table S3 **Nucleotide sequence (5′ to 3′) of individual CRISPR spacers identified in this study.**
(DOCX)

Table S4 **CRISPR array genotype of individual** ***E. amylovora*** **strains examined in this study.**
(DOCX)
