# Supplementary figures and images for: Erwinia amylovora CRISPR Elements Provide New Tools for Evaluating Strain Diversity and for Microbial Source Tracking
Source: PLoS One. 2012 Jul 31;7(7):e41706. doi: 10.1371/journal.pone.0041706 (PMC3409226; doi:10.1371/journal.pone.0041706)

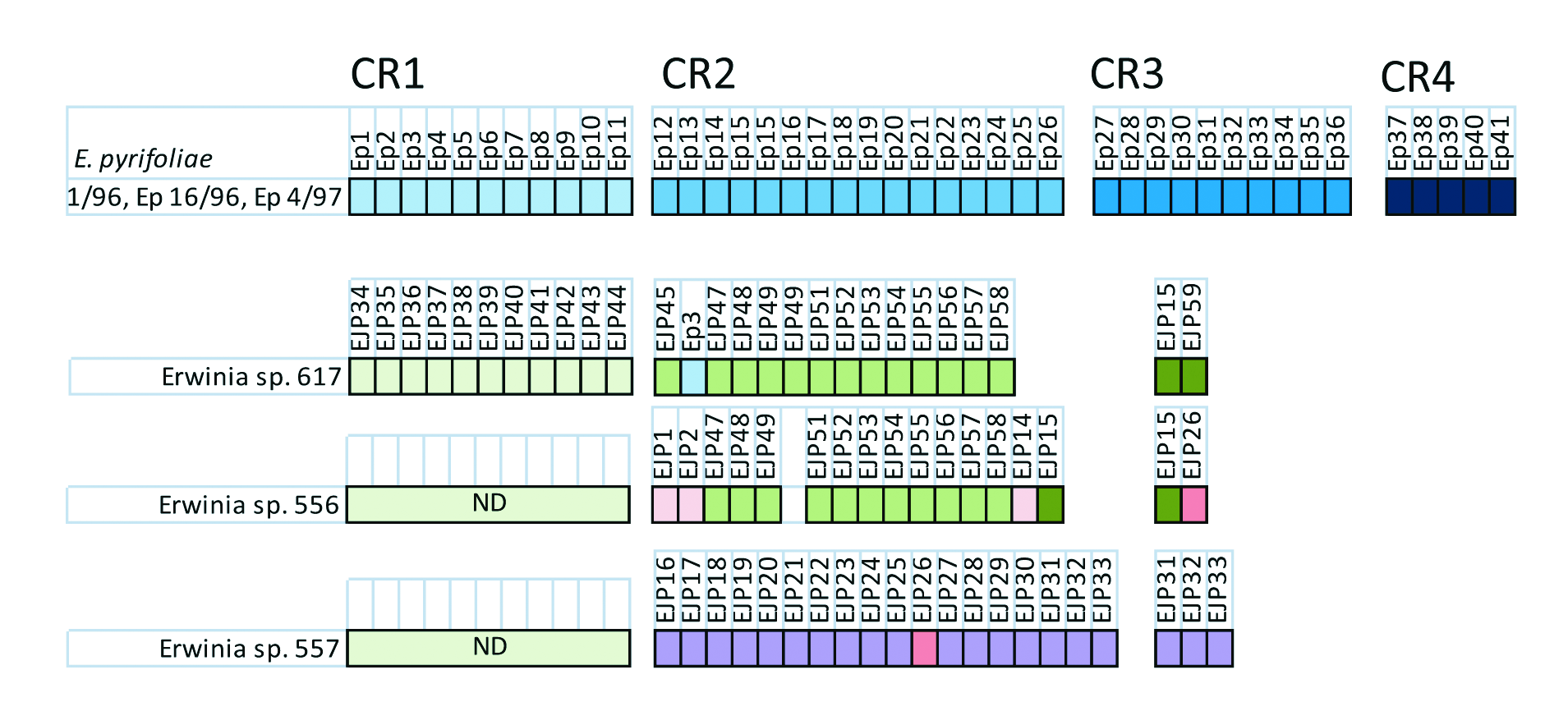

Supplement: Figure S1 — Graphic representation of spacers grouped into patterns from CRISPR arrays CR1, CR2, and CR3 of E. pyrifoliae and Erwinia sp. strains. Individual spacer sequences are represented by boxes; spacers were considered unique if they contained >5 nucleotide differences compared to other spacer sequences. Each unique spacer was assigned a number with an Ep (E. pyrifoliae) or EJP (Erwinia sp.) prefix. Only spacer Ep3 was shared among E. pyrifoliae and Erwinia sp. (strain 617). Empty areas indicate the corresponding spacer in other similar patterns is not present. ND indicates that the sequence of CR1 was not determined for Erwinia sp. 556 and 557. (TIF) [file pone.0041706.s001.tif]
