# Supplementary material for: Erwinia amylovora CRISPR Elements Provide New Tools for Evaluating Strain Diversity and for Microbial Source Tracking
Source: PLoS One. 2012 Jul 31;7(7):e41706. doi: 10.1371/journal.pone.0041706 (PMC3409226; doi:10.1371/journal.pone.0041706)
Supplement: Table S1 — Oligonucleotide primers used in this study and their description. (DOCX) [file pone.0041706.s002.docx]

Table S1. Oligonucleotide primers used in this study and their description.

| **Primer** | **Sequence (5’ – 3’)** | **Description** |
| --- | --- | --- |
| CR1-F1 | CGCCGCCACGCTGCCATTT | Forward primer for amplification and partial sequencing of *E. amylovora* CRISPR1 from apple, pear and quince hosts. This study |
| C1-R0 | TCCAGCGCCTGTAAAGCGGC | Reverse primer for amplification and partial sequencing of *E. amylovora* CRISPR1 from apple, pear and quince hosts. Flanks CRISPR1 region. This study |
| C1Uni-F | AGCCGAYTTTYCCYGYTTTRAG | Forward primer for amplification and partial sequencing of CRISPR1 from *E. amylovora* *Rubus* isolates. Can also be used as a universal primer for forward amplification. This study |
| CR1Ru-R | CGTCAGTGGAGTATGCTTTGGTTT | Reverse primer for amplification and partial sequencing of CRISPR1 from *E. amylovora* *Rubus* isolates. This study |
| CR1-F2 | ATAAACCGCAAGCGATCAACCTGT | Forward primer used to sequence CRISPR1 (aligns to spacer 6, partial). This study |
| CR1-F3 | GCTTATACAACTGACAAAATCGTG | Forward primer used to sequence CRISPR1 (aligns to spacer 15, partial). This study |
| CR1RevRpt | CGGTTTATCCCCGCTCACGC | Reverse primer that binds to repeats of CRISPR1. This study |
| Cr2-F1 | GCGGCCAACAGATGCGGAAAG | Forward primer for amplification and partial sequencing of *E. amylovora* CRISPR2 for all hosts. This study |
| Cr2-F2 | GTCTGGCGCAAAAACTGGAG | Forward primer used to sequence CRISPR2 (aligns to spacer 36, partial). This study |
| Cr2-F3 | CCGCCCTTCTGGTGTTTTGA | Forward primer used to sequence CRISPR2 (aligns to spacer 47, partial). This study |
| Cr2-R2 | ACACGTGGTTTCTGAGTCTGGA | Reverse primer used to sequence CRISPR2 (aligns to spacer 59, partial). This study |
| C2-R1 | TGCGGGGAACACTCGACATCTAAT | Reverse primer for amplification and partial sequencing of *E. amylovora* CRISPR2 for all hosts. This study |
| CR3-F1 | TTTTCGCCGGGTAACAGG | Forward primer for amplification and partial sequencing of *E. amylovora* CRISPR3 for all hosts. This study |
| CR3-R1 | ATGAGAAGCCCGTGAAGCAAAGTA | Reverse primer for amplification and partial sequencing of *E. amylovora* CRISPR3 for all hosts. This study |
| EPyF-1 | GCAAAGCGGCCACCAACC | Forward primer for amplification and partial sequencing of CRISPR1 in *E. pyrifoliae*. This study |
| EPyR-1 | GGCGCGTACCGGTTGTTAGCAG | Reverse primer for amplification and partial sequencing of CRISPR1 in *E. pyrifoliae* . This study |
| EPyF-2 | GGTCTGCGGGCTGGTGTCATTTT | Forward primer for amplification and partial sequencing of CRISPR2 in *E. pyrifoliae* and Erwinia from Japan |
| EPyR-2 | GAGGCCCGCTTAGCATCTGTCA | Reverse primer for amplification and partial sequencing of CRISPR2 in *E. pyrifoliae* and Erwinia from Japan |
| EpyF-3 | ATTTATATCGCGTGGTTGTC | Forward primer for amplification and partial sequencing of CRISPR3 in *E. pyrifoliae*. This study |
| EpyR-3 | TGATGATATTGGCTGGAAAGT | Reverse primer for amplification and partial sequencing of CRISPR3 in *E. pyrifoliae.* This study |
| EpyF-4 | AGGTGGCGTTAGCATAGCGGT | Forward primer for amplification and partial sequencing of CRISPR4 in *E. pyrifoliae*. This study |
| Epy4R-4 | CACTCAGGCAAGGGAATGGGT | Reverse primer for amplification and partial sequencing of CRISPR4 in *E. pyrifoliae*. This study |
| groEL-A | GAAGTKGCCTCTAAAGCGAATGA | PCR amplification and sequencing of partial *groEL* fragment. Forward primer. McGhee et al, 2002 (22) |
| groEL- B | GCMACRCCACCACCAGCAACC | PCR amplification and sequencing of partial *groEL* fragment. Reverse primer. McGhee et al, 2002 (22) |
