# Supplementary material for: Erwinia amylovora CRISPR Elements Provide New Tools for Evaluating Strain Diversity and for Microbial Source Tracking
Source: PLoS One. 2012 Jul 31;7(7):e41706. doi: 10.1371/journal.pone.0041706 (PMC3409226; doi:10.1371/journal.pone.0041706)
Supplement: Table S2 — GenBank accession numbers of CRISPR spacer array sequences CR1, CR2, and CR3 for each E. amylovora strain determined in this study. (DOCX) [file pone.0041706.s003.docx]

Table S2. GenBank accession numbers of CRISPR spacer array sequences CR1, CR2, and CR3 for each *E. amylovora* strain determined in this study.

| Strain | CRISPR1 | CRISPR2 | CRISPR 3 | Strain | CRISPR1 | | CRISPR2 | | CRISPR 3 |
| --- | --- | --- | --- | --- | --- | --- | --- | --- | --- |
| **Midwest, Eastern US and Canada - Sm^S^** | | | | | | | | | |
| 6-97 | JQ796405 | JQ796474 | JQ796562 | K2 | JQ796420 | | JQ796488 | | JQ796580 |
| BH | JQ796399 | JQ796467 | JQ796556 | L14 | JQ796427 | | JQ796493 | | JQ796587 |
| DP11 | JQ796407 | JQ796476 | JQ796564 | MK1 | JQ796431 | | JQ796497 | | JQ796591 |
| DR5 | JQ796408 | JQ796477 | JQ796565 | NW17-4 | JQ796459 | | JQ796537 | | JQ796624 |
| Ea110 | JQ796409 | JQ796479 | JQ796567 | Pn | JQ796436 | | JQ796502 | | JQ796597 |
| Ea273 | JQ796462 | JQ796547 | JQ796630 | RB02 | JQ796437 | | JQ796503 | | JQ796599 |
| Ea(T1)2 | JQ796448 | JQ796514 | JQ796610 | RB07 | JQ796438 | | JQ796504 | | JQ796600 |
| Ea(T3)2 | JQ796449 | JQ796515 | JQ796609 | RL3 | JQ796441 | | JQ796507 | | JQ796603 |
| EL01 | JQ796412 | JQ796481 | JQ796570 | RRP12 | JQ796445 | | JQ796511 | | JQ796606 |
| GH9 | JQ796414 | JQ796482 | JQ796572 |  |  | |  | |  |
| **Midwest, Eastern US and Canada – Sm^R^** | | | | | | | | | |
| BBA-8 | JQ796402 | JQ796470 | JQ796557 | NW1-1 | JQ796455 | | JQ796533 | | JQ796621 |
| BCN20 | JQ796401 | JQ796469 | JQ796558 | NW18-6 | JQ796460 | | JQ796538 | | JQ796625 |
| CA11 | JQ796404 | JQ796473 | JQ796561 | NW2A | JQ796457 | | JQ796535 | | JQ796627 |
| DM1 | JQ796406 | JQ796475 | JQ796563 | NW2-1 | JQ796456 | | JQ796534 | | JQ796622 |
| EaRoo29 | JQ796444 | JQ796510 | JQ796598 | NW3-1 | JQ796458 | | JQ796536 | | JQ796623 |
| GR5B1 | JQ796415 | JQ796483 | JQ796573 | NW21-4 | JQ796461 | | JQ796539 | | JQ796626 |
| HS10 | JQ796416 | JQ796484 | JQ796574 | NW H26 | JQ796381 | | JQ796540 | | JQ796628 |
| KL | JQ796421 | JQ796489 | JQ796581 | RA | JQ796443 | | JQ796509 | | JQ796605 |
| KR | JQ796422 | JQ796490 | JQ796582 | RM5 | JQ796442 | | JQ796508 | | JQ796604 |
| MA-1 | JQ796428 | JQ796494 | JQ796588 | SB1-9 | JQ796447 | | JQ796513 | | JQ796608 |
| MC-5 | JQ796429 | JQ796495 | JQ796589 | S5 | JQ796446 | | JQ796512 | | JQ796607 |
| MI5-1 | JQ796430 | JQ796496 | JQ796590 | W4 | JQ796453 | | JQ796518 | | JQ796614 |
| **Western US** | | | | **Europe, Middle East, and New Zealand** | | | | | |
| 87-70 | JQ796394 | JQ796463 | JQ796548 | 1596 | | JQ796396 | JQ796464 | JQ796550 | |
| 87-73 | JQ796395 | JQ796521 | JQ796549 | B3 | | JQ796400 | JQ796468 | JQ796555 | |
| Ca1R | JQ796378 | JQ796471 | JQ796559 | CFBP1430 | | JQ796379 | JQ796531 | JQ796631 | |
| Ca3R | JQ796403 | JQ796472 | JQ796560 | Ea1189 | | JQ796411 | JQ796480 | JQ796569 | |
| Ea88 | JQ796380 | JQ796478 | JQ796566 | Ea322 | | JQ796410 | JQ796519 | JQ796568 | |
| FB93-9 | JQ796413 | JQ796523 | JQ796571 | Leb A-1 | | JQ796424 | JQ796525 | JQ796551 | |
| JL1189 | JQ796383 | JQ796530 | JQ796579 | Leb A-16 | | JQ796425 | JQ796491 | JQ796553 | |
| LA004 | JQ796384 | JQ796527 | JQ796583 | Leb A-19 | | JQ796426 | JQ796465 | JQ796554 | |
| La092 | JQ796423 | JQ796528 | JQ796584 | Leb A-3 | | JQ796397 | JQ796520 | JQ796552 | |
| LP101 | JQ796385 | JQ796492 | JQ796586 | Leb B-66 | | JQ796398 | JQ796466 | JQ796585 | |
| OR1 | JQ796388 | JQ796499 | JQ796594 | NZR3 | | JQ796386 | JQ796541 | JQ796617 | |
| OR6 | JQ796389 | JQ796500 | JQ796595 | NZR5 | | JQ796433 | JQ796498 | JQ796592 | |
| UTRJ2 | JQ796452 | JQ796517 | JQ796612 | NZS24 | | JQ796387 | JQ796542 | JQ796616 | |
| UT5P4 | JQ796450 | JQ796516 | JQ796613 | OT-1 | | JQ796435 | JQ796501 | JQ796596 | |
| UTFer3 | JQ796451 | JQ796522 | JQ796611 |  | |  |  |  | |
| WSDA 16 | JQ796454 | JQ796529 | JQ796615 |  | |  |  |  | |
| ***E. amylovora* strains from alternate hosts** | | | | | | | | | |
| IH2-3 | JQ796417 | JQ796485 | JQ796575 | RBA4 | | JQ796439 | JQ796505 | JQ796601 | |
| IH3-1 | JQ796418 | JQ796486 | JQ796576 | Rkk3 | | JQ796440 | JQ796506 | JQ796602 | |
| IL5 | JQ796382 | JQ796526 | JQ796577 | TxLo3 | | JQ796390 | JQ796543 | JQ796618 | |
| IL6 | JQ796419 | JQ796487 | JQ796578 | TxLo4 | | JQ796391 | JQ796544 | JQ796619 | |
| MR1 | JQ796432 | JQ796532 | JQ796632 | TxLo6 | | JQ796392 | JQ796545 | JQ796620 | |
| OKR1 | JQ796434 | JQ796524 | JQ796593 | TxLo7 | | JQ796393 | JQ796546 | JQ796629 | |
