# Supplementary material for: Erwinia amylovora CRISPR Elements Provide New Tools for Evaluating Strain Diversity and for Microbial Source Tracking
Source: PLoS One. 2012 Jul 31;7(7):e41706. doi: 10.1371/journal.pone.0041706 (PMC3409226; doi:10.1371/journal.pone.0041706)
Supplement: Table S3 — Nucleotide sequence (5′ to 3′) of individual CRISPR spacers identified in this study. (DOCX) [file pone.0041706.s004.docx]

Table S3. Nucleotide sequence (5’ to 3’) of individual CRISPR spacers identified in this study.

Supplementary table 3 page 2 of 6

Supplementary table 3 page 3 of 6

Supplementary Table 3 page 4 of 6

Supplementary Table 3 page 5 of 6

Supplementary Table 3 page 6 of 6.
