# Supplementary material for: Erwinia amylovora CRISPR Elements Provide New Tools for Evaluating Strain Diversity and for Microbial Source Tracking
Source: PLoS One. 2012 Jul 31;7(7):e41706. doi: 10.1371/journal.pone.0041706 (PMC3409226; doi:10.1371/journal.pone.0041706)
Supplement: Table S4 — CRISPR array genotype of individual E. amylovora strains examined in this study. (DOCX) [file pone.0041706.s005.docx]

Table S4. CRISPR array genotype of individual *E. amylovora* strains examined in this study.

| Host and CRISPR genotype | Strains from this study |
| --- | --- |
| **Apple/pear/quince** |  |
| 1-21-38 | Ea273, |
| 2-22-38 | BH, Pn, 6-97, NW 17-4 |
| 3-23-38 | RA |
| 3-24-38 | GH9, DR5, DP11 |
| 4-22-38 | Ea(T3)2, Ea(T1)2, WSDA16 |
| 4-23-38 | Ea110, MA-1, MC-5, W4, BBA-8, RM5, KR, SB1-9, MI5-1, CA11, BCN20, DM1, GR5B1, HS10, KL, EaRoo 29, NW2A, NW2-11, NW18-6, NW21-4, NW H26 |
| 4-24-38 | RRP12, Ea1189, CFBP1430, B66, A16, LebA-1, 1596, B3, OT-1 |
| 4-25-38 | MK1 |
| 4-27-38 | EL01, K2, RB02, S5 |
| 4-28-38 | RB07 |
| 5-24-38 | 87-70, 87-73, EA322, LebA-3, LebA-19, NZR5, NZR3, NZS24, |
| 5-27-38 | RL3 |
| 6-24-38 | L14 |
| 7-24-38 | FB93-9 |
| 7-29-38 | UTRJ2, UT5P4, UTFer3 |
| 8-32-38 | CA3R |
| 9-23-38 | NW1-1, NW3-1 |
| 12-34-38 | OR1, CA1R, LP101 |
| 13-34-38 | La004 |
| 14-34-38 | OR6 |
| 15-34-38 | Ea88, JL1189, La092 |
|  |  |
| **Indian Hawthorn**  10-37-38 | IH2-3, IH3-1 |
| **Loquat** 11-26-38 | TxLo3, TxLo4, TxLo6, TxLo7 |
| **Rubus** 16-30-39 | IL5, IL6 |
| **Rubus** 17-36-39 | MR1 |
| **Rubus** 18-31-39 | OKR1 |
| **Rubus** 19-35-39 | RBA4 |
| Rubus 20-33-39 | RKK3 |
